# Sonographic Characteristics and Pathology Correlation of Breast Imaging Reporting and Data System (BI-RADS) Category 4 Lesions

**DOI:** 10.7759/cureus.51410

**Published:** 2023-12-31

**Authors:** Poonum Khan, Imrana Masroor, Muhammad S Alam, Abdus Salam, Yasir Ali, Muhammad Salman Khan

**Affiliations:** 1 Radiology, Aga Khan University, Karachi, PAK; 2 Radiology, King Faisal Specialist Hospital and Research Centre, Medina, SAU; 3 Internal Medicine, Khyber Teaching Hospital, Peshawar, PAK; 4 Internal Medicine, Nazareth Hospital, Philadelphia, USA; 5 Diagnostic and Interventional Imaging, University of Texas Health Science Center at Houston, Houston, USA

**Keywords:** ultrasound-guided biopsy, histopathology, breast cancer diagnosis, mammography, bi-rads 4

## Abstract

Introduction: The Breast Imaging-Reporting and Database System (BI-RADS) category 4 is designated for breast lumps that do not display the typical features of malignancy but still raise enough suspicion to warrant a recommendation for a biopsy, as malignancy cannot be ruled out through imaging alone. The main objective of this study was to investigate the sonographic characteristics and pathology correlation of BI-RADS 4 breast lesions and determine the positive predictive rate of BI-RADS 4 lesions in diagnosing breast cancer, using histopathology as the gold standard.

Methods: This was a cross-sectional study conducted at the Department of Radiology, Aga Khan University Hospital in Karachi, spanning from May 2021 to August 2022, with a duration of 15 months. The study focused on female patients over the age of 18 who presented with suspicious breast lesions on ultrasound. Both mammography and ultrasound-guided core needle biopsy were performed on these patients, followed by a detailed histopathological evaluation of the biopsy specimens. To calculate the positive predictive value (PPV), true positive cases were identified through both histopathology and ultrasonography.

Results: A total of 227 cases were categorized as BI-RADS 4 lesions, with the patients' mean age being 47.8 ± 14.3 years (range: 17 - 88). Among the biopsied lesions, 101 cases were confirmed to be true positive for breast malignancies, resulting in a PPV for malignancy of 44.9%. Conversely, there were 124 false positive cases out of the 227 BI-RADS 4 category lesions (54.63%). The primary indication for presentation was a breast lump, and out of the 101 confirmed malignant cases, 70 (69.3%) were associated with malignancy.

Conclusion: BI-RADS 4 can be utilized to assess suspicious breast lumps; however, for more reliable results and to avoid false negatives, histopathological confirmation should complement the imaging findings.

## Introduction

Breast cancer is the most prevalent form of cancer and a leading cause of cancer-related deaths among women worldwide. The reduction in breast cancer mortality heavily relies on early detection and appropriate treatment [1]. In Pakistan, breast cancer affects one in every nine Pakistani women, resulting in one of the highest incidence rates in Asia [2,3]. Recent data from the Shaukat Khanum Memorial Cancer Hospital indicate that the incidence of breast cancer in Pakistan is 21.5% among all patients and 45.9% among female patients [3,4].

Mammography serves as the primary imaging modality for breast cancer screening and diagnosis. Numerous randomized controlled studies have demonstrated an average 30% reduction in mortality through screening women over 50 years of age [5]. Additionally, screening has shown a 16% reduction in breast cancer mortality among women aged 39-49 years [6].

Percutaneous imaging-guided breast biopsy has emerged as a reliable alternative to surgical biopsy for obtaining a histological diagnosis. This less invasive procedure offers advantages such as quick performance, fewer complications (such as hematoma and infection), and cost-effectiveness [7].

To standardize mammography reporting, clarify interpretations, and enhance communication between clinicians, the American College of Radiology (ACR) developed the Breast Imaging-Reporting and Database System (BI-RADS) in 1993 [8,9]. Furthermore, BI-RADS has demonstrated improvements in the positive predictive value (PPV) of breast biopsies [10]. Acknowledging the increased use of breast sonography, the ACR recently introduced a BI-RADS lexicon for breast sonography to standardize lesion characterization [11].

Within the BI-RADS classification, category 4 is reserved for findings that do not exhibit the classic appearance of malignancy but still raise enough suspicion to warrant a recommendation for biopsy, as malignancy cannot be excluded based on imaging alone. Category 3 assessments have a ceiling of 2% likelihood of malignancy, while category 5 assessments have a floor of 95% [11,12].

Few studies have examined the PPV of category 4 lesions. Zonderland et al. conducted a study revealing carcinoma in 39 (52.7%) out of 74 category 4 lesions [12], and another study by Orel et al. reported a PPV of 30% for BI-RADS 4 lesions [10]. Given the wide range of malignancy likelihood in category 4 breast lesions, these findings pose significant challenges for clinicians when making diagnostic decisions.

In our investigation, we conducted an extensive literature search on PubMed, PubMed Central, Google Scholar, Scopus, Embase, and PakMedinet to identify regional and local studies on this subject matter. However, to the best of our knowledge, no studies have assessed the PPV of BI-RADS 4 lesions in Pakistan. Therefore, the aim of this study was to investigate the sonographic characteristics and pathology correlation of BI-RADS 4 breast lesions and to determine the proportion of breast lesions classified as BI-RADS 4, according to ACR guidelines, that are confirmed as malignant through biopsy, with histopathology serving as the gold standard. We anticipate that the results of this study will provide patients and referring clinicians with valuable information to facilitate informed decision-making regarding the appropriate course of action for such breast lesions.

## Materials and methods

Study design, setting, and participants

The study was conducted at the Department of Radiology, Aga Khan University Hospital, Karachi, Pakistan. The study duration was six months following the approval. It was a cross-sectional study with a sample size calculated using OpenEpi®. The sample size of 227 was determined based on a PPV of 30.0% for BI-RADS 4 lesions in detecting breast cancer, a 95% confidence interval, and a margin of error of 6%. Non-probability consecutive sampling was used to select participants. The study included all female patients above the age of 18 with a breast lump measuring 1 mm or greater. Suspicious breast lumps were characterized by oval, well-circumscribed, hypoechoic solid masses, as well as irregular masses with heterogeneous echo texture and posterior acoustic shadowing. These findings did not necessarily have the classic appearance of malignancy but were sufficiently suspicious to warrant a recommendation for biopsy according to ACR guidelines. Patients with incomplete medical records, those lost to follow-up, and those who did not undergo breast lump biopsies were excluded from the study. The Ethics Review Committee (ERC) at Aga Khan University Hospital in Karachi, Pakistan, granted ethical approval for this study (Ref No: 4219-Rad-ERC-21).

Data collection

The study was conducted after approval from the Ethics Review Committee, Aga Khan University, Pakistan. Informed consent was obtained from all patients undergoing breast biopsies after explaining the details of the associated benefits and risks of the relevant procedures. Mammography was performed using Mammomat 3000 NOVA (Siemens, Munich, Germany), obtaining images in two standard views (mediolateral oblique and craniocaudal), with additional views as necessary. Additional ultrasound was performed if needed. Mammogram and sonogram image interpretation were conducted by radiologists with at least five years of experience, following the guidelines of the ACR.

All lesions categorized as BI-RADS 4 on mammogram or ultrasound were included in the study and underwent ultrasound-guided core needle biopsy. The core needle biopsies were performed by dedicated radiologists with at least five years of experience under ultrasound guidance (Xario 200; Toshiba, Tokyo, Japan) using a 14-gauge biopsy needle. All core biopsy specimens were sent for histopathology.

The data collected included patient age, family history of breast cancer, personal history of breast cancer, size, side of the lesion (right/left breast), morphological features of suspicious abnormality, and final histopathology report.

Data analysis

Statistical analysis was conducted using the Statistical Package for Social Sciences (SPSS) version 26.0 (IBM Corp., Armonk, NY, USA). Continuous data were summarized as mean (SD) or median (range) as appropriate. Categorical data were summarized as counts and percentages. The biopsy rate was determined from all patients categorized in the BI-RADS 4 category. The true positive rate was calculated by dividing the total number of malignant biopsy results by the total number of BI-RADS 4 lesions.

## Results

**Figure 1:**
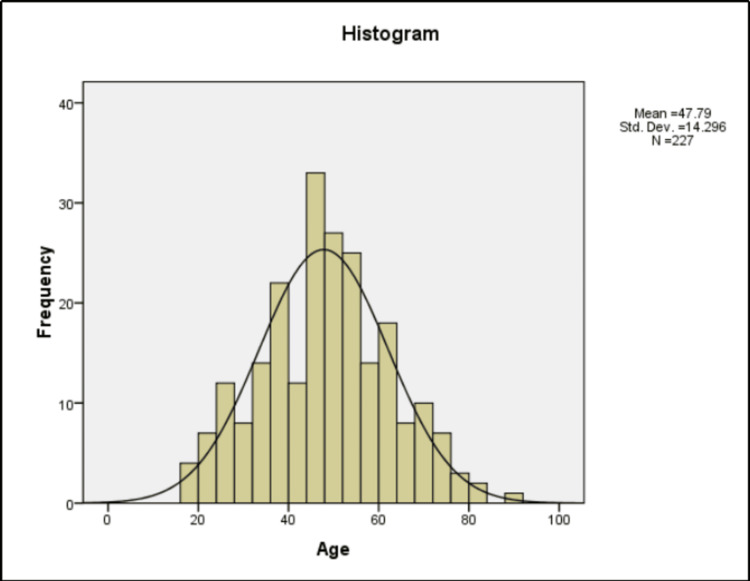
Age distribution of patients Std. Dev. = Standard Deviation, N = Sample size

Between May 2021 and August 2022, 227 breast lesions were categorized in BI-RADS category 4. The mean age of the patients at initial imaging was 47.8 years (standard deviation, 14.3 years), with an age range of 17 to 88 years. The details of the age distribution of the patients are given in Figure 1.

Fifteen out of 227 patients (6.61%) had a personal history of breast cancer. Palpable masses were noted in 148 cases (65.20%). A family history of breast cancer was observed in 11 cases (4.85%). History of reddish nipple discharge was seen in 12 cases (5.29%). Mastalgia was reported by 13 (5.73%) patients. Nipple discharge, skin changes, and Paget’s disease of the breast were reported by 12 (5.29%), three (1.32%), and one (0.44%) patients, respectively. The details of indications for mammography are listed in Table 1.

**Table 1:** Indications for mammography n = Frequency, % = Percentage

Indication of Mammography	Frequency (n)	Percentage (%)
History of Breast Cancer	15	6.61
Family History of Breast Cancer	11	4.85
Breast Lump	148	65.20
Mastalgia	13	5.73
No Complaints	5	2.20
Nipple Discharge	12	5.29
Skin Changes	3	1.32
Paget’s Disease	1	0.44
Routine Examination	12	5.29
Screening Examination	7	3.08

As summarized in Table 2, there were 101 true positive cases out of 227 biopsied lesions (44.9% PPV for malignancy) on histopathology. The false positive cases accounted for 124 out of 227 lesions (54.63%). The biopsy results were inconclusive for two patients. Table 3 displays biopsy results in relation to presenting complaints.

**Table 2:** True positive and false positive values based on biopsy report n = Frequency, % = Percentage

Biopsy Report	Frequency (n)	Percentage (%)
Benign (False Positive)	124	54.63
Malignant (True Positive)	101	44.49
Inconclusive	2	0.88

**Table 3:** Biopsy results in relation to initial mammography indication n = Frequency, % = Percentage

Indications	Benign n (%)	Malignant n (%)
History of Breast Carcinoma	7 (5.65)	7 (6.93)
			Family History of Breast Carcinoma	8 (6.45)	3 (2.97)
Breast Lump	78 (62.90)	70 (69.31)			
			Mastalgia	8 (6.45)	4 (3.96)
No Complaints	3 (2.42)	2 (1.98)			
			Nipple Discharge	7 (5.65)	5 (4.95)
Skin Changes	0 (0)	3 (2.97)			
			Paget’s Disease of Breast	0 (0)	1 (0.99)
Routine Examination	11 (8.87)	1 (0.99)			
			Screening	2 (1.61)	5 (4.95)

The most common benign pathology was fibroadenoma, observed in 57 cases (25%). The most common malignant pathology was intraductal carcinoma (IDC), seen in 89 cases (39.21%). Unspecified benign tissue, intraductal papilloma, and ductal hyperplasia were observed in 22 (9.69%), 16 (7.05%), and nine (3.96%) patients, respectively. The details of histopathology results are provided in Table 4. Table 5 shows rates of benign and malignant BI-RADS 4 lesions in terms of ultrasonography (US) lexicon descriptors.

**Table 4:** Histopathology findings of the needle biopsy n = Frequency, % = Percentage

HISTOPATHOLOGICAL FINDINGS	Frequency (n)	Percentage (%)
Benign Tissue (Not Specified)	22	9.69
Chronic Inflammation	1	0.44
Ductal Carcinoma In Situ	4	1.76
Ductal Hyperplasia	9	3.96
Fibroadenoma	57	25.11
Intraductal Carcinoma	89	39.21
Inconclusive	2	0.88
Inflammatory	3	1.32
Intraductal Papilloma	16	7.05
Invasive Carcinoma	3	1.32
Malignant Phylloides	1	0.44
Papillary Ductal Carcinoma	5	2.20
Phylloides	5	2.20
Sclerosis Adenosis	6	2.64
Stromal Sclerosis	4	1.76

**Table 5:** Rates of benign and malignant BI-RADS 4 lesions in terms of US lexicon descriptors n = Frequency, % = Percentage, US = Ultrasonography

Descriptors			Biopsy Result		
			Benign	Inconclusive	Malignant
Shape	Elongated	Count	28	0	10
		% within Shape	73.7	0	26.3
	ILLDEFINED	Count	32	0	67
		% within Shape	32.3	0	67.7
	OVAL	Count	4	0	2
		% within Shape	66.7	0	33.3
	ROUNDED	Count	60	2	22
		% within Shape	71.4	2.4	26.2
Pleomorphic calcifications	Absent	Count	80	1	73
		% within Pleomorphic calcifications	51.9	0.6	47.4
	Not Done	Count	38	1	13
		% within Pleomorphic calcifications	73.1	1.9	25
	Present	Count	6	0	15
		% within Pleomorphic calcifications	28.6	0	71.4
Margins	Irregular	Count	35	0	75
		% within Margins	31.8	0	68.2
	Lobulated	Count	17	0	1
		% within Margins	94.4	0	5.6
	Regular	Count	72	2	25
		% within Margins	72.7	2	25.3

## Discussion

The advancement of US equipment in recent times has significantly enhanced the efficacy of US in breast imaging [8]. Particularly in women under the age of 50, the detection of mammographically occult masses through US has risen by up to 27% [8]. To standardize the characterization of breast lesions with US, mirroring mammography, the American College of Radiology introduced the first version of the Breast Imaging Reporting and Data System (BI-RADS) US lexicon in 2003 [13,14]. Subsequently, the second version of the BI-RADS US lexicon was published in the fifth edition of the BI-RADS atlas in 2013 [13-15].

The initial BI-RADS US lexicon included descriptors such as shape, orientation, margins, lesion boundary, echo pattern, posterior acoustic features, and surrounding tissue alterations [8]. The second version brought minimal changes, retaining descriptors like shape, orientation, margin, and features, but eliminating the lesion boundary. Nomenclature adjustments included "posterior features" replacing "posterior acoustic features" and "associated features" replacing "surrounding tissue alterations." Additionally, the second version introduced "elasticity assessment" among the associated features and added the term "heterogeneous" to the echo pattern. Utilizing these descriptors, each lesion is assigned a final assessment category, aligning with the mammography model, ranging from 0 to 6 [12,13]. Specifically, BI-RADS 4 denotes findings lacking the classic appearance of malignancy but warranting a biopsy recommendation. Category 3 indicates a 2% likelihood of malignancy, while category 5 represents a 95% likelihood, with category 4 encompassing a broad range in between [13-15].

To enhance internal audits, communication with clinicians, pathologists, and image-directed research, many facilities further subdivide category 4 into 4A, 4B, and 4C. Despite the paucity of studies evaluating the pathological results of these subcategories and their PPVs [12,10], our study aimed to calculate the PPV of BI-RADS 4 and its subcategories (4A, 4B, 4C) for breast cancer, while also assessing the correlation between BI-RADS US lexicon descriptors and pathology results.

Lesions falling under the BI-RADS 4 category pose a higher risk of malignancy ("suspicious lesion"), necessitating biopsy consideration. In our study, 227 lesions were biopsied; however, BIRADS 4 lesions were not further classified into subcategories 4A, 4B, and 4C, indicating an unexplored gradient of increasing malignancy risk. Previous studies on PPV have shown a wide range, from 6.2% to 52.7% [10,12,14], likely influenced by variations in breast cancer prevalence and patient selection criteria.

Several reports have explored the PPV of BI-RADS for mammography and US, analyzing lesion descriptors and final assessment categories. Liberman et al. [15] found that the BI-RADS lexicon for mammography enabled the quantification of carcinoma likelihood, with spiculated margins and irregular shape strongly associated with malignant masses. Burnside et al. [16] and Bent et al. [17] similarly concluded that BI-RADS morphology and distribution descriptors aid in assessing malignancy risk.

Our study, with an overall PPV for malignancy in BIRADS category 4 images of 44.49% (101 of 227 patients), aligns with the range observed in previous studies. Clinically palpable masses exhibit a significantly higher prevalence of malignancy than other clinical findings, constituting the most common observation in mammography and sonography. Invasive ductal carcinoma emerged as the predominant malignancy, while fibroadenoma was the prevalent benign pathology.

Examining lesion margins, our study revealed irregular borders in 68.2% of malignant lesions compared to regular margins in 72% of benign lesions. Lesion shape emerged as a significant criterion for differentiation, with our study demonstrating a significant difference (P < 0.05) between benign and malignant lesions. Oval and round-shaped lesions were more often benign, while irregular-shaped lesions were more often malignant.

Pleomorphic calcifications were identified as an important indicator of malignancy in our study, with 73% of malignant lesions exhibiting this feature. However, our study did not categorize lesions into 4A, 4B, and 4C subcategories, representing a significant limitation. Subcategorization, reliant on the radiologist's experience, can improve internal audits, communication, and research but demands clear and objective rules, particularly challenging for smaller lesions.

## Conclusions

Our study underscores the valuable role of the BI-RADS 4 category in assessing the likelihood of malignancy in breast lesions, with an overall PPV of 44.49%. Notably, the absence of subcategorization (4A, 4B, 4C) in our analysis limits a nuanced understanding of increasing malignancy risks within this category. Lesion characteristics, such as irregular margins, shape, and the presence of pleomorphic calcifications, proved significant in distinguishing between benign and malignant lesions. The findings emphasize the importance of standardized descriptors and subcategorization to enhance the precision of risk assessment, ultimately aiding clinicians, pathologists, and researchers in making informed decisions. Further research with comprehensive subcategorization is warranted to enhance the utility of BI-RADS 4 in clinical practice.
